# The Influence of Pregnancy on the Recurrence of Cutaneous Malignant Melanoma in Women

**DOI:** 10.1155/2010/214745

**Published:** 2010-08-01

**Authors:** M. Albersen, V. I. Westerling, P. A. M. van Leeuwen

**Affiliations:** ^1^Department of Metabolic and Endocrine Diseases, Wilhelmina Children's Hospital, University Medical Centre Utrecht, 3584 EA Utrecht, The Netherlands; ^2^Department of General Practice, University Medical Centre Utrecht, 3732 HJ De Bilt, The Netherlands; ^3^Department of Surgical Oncology, VU University Medical Centre, 1081 HV Amsterdam, The Netherlands

## Abstract

*Objective*. The aim of this study was to determine whether pregnancy increases the recurrence risk of cutaneous malignant melanoma (CMM) in women with a history of stage I CMM. *Methods*. The electronic medical databases of Medline and Embase were explored. All 1084 obtained articles were screened on title and abstract using predetermined inclusion and exclusion criteria. A critical appraisal of relevance and validity was conducted on the remaining full text available articles. *Results*. Two studies were selected. Both studies revealed no significant difference in disease-free survival between women with stage I CMM and the control population. *Conclusion*. Pregnancy does not increase the recurrence risk of CMM in women with a history of stage I CMM.

## 1. Clinical Scenario

A 33-year-old nulliparous woman was seen by the dermatologist because of a mole on her leg, which had been growing and itching over the past year. After excision of the lesion and pathological examination, the diagnosis made was a cutaneous malignant melanoma, with Clark level IV, a Breslow thickness of 1.25 cm-and tumor-free margins. Because excision had been successful, only a sentinel lymph node dissection was conducted which yielded no metastatic spread. The conclusive diagnosis therefore was a stage I cutaneous malignant melanoma. Every three months now, the patient is seen by the dermatologist in order to keep a close watch on the development of recurrence. Currently, she and her partner would like to embark on a first pregnancy. 

## 2. Introduction

The incidence of Cutaneous Malignant Melanoma (CMM) has shown a rapid increase over the past decades, therefore substantiating a growing problem. About 27% of the noninvasive CMM diagnosed in the Netherlands in 2003 were seen in women between 30 and 44 years of age, of which 13% eventually died because of the disease. The currently known factors determining outcome are mostly the skin location of the melanoma, the Breslow thickness, and the Clark level [1, 2]. Keeping in view the possibility of tumor sensitivity to hormones, although the possible mechanism of this influence is still unknown, the question arises whether pregnancy influences the recurrence of CMM [2]. In the past, some studies have suggested a negative influence [3, 4], whereas other studies showed no effect of pregnancy on the prognosis of cutaneous malignant melanoma [5, 6]. Therefore, the aim of this article is to answer the following clinical question: 

does [pregnancy] increase the [recurrence) risk (of cutaneous malignant melanoma] in [women with a history of stage I cutaneous malignant melanoma]?

Pregnancy was defined as occurring within five years of CMM diagnosis in previously nulliparous women. Stage I cutaneous melanoma was defined as a malignant melanoma localized to the site of origin on the skin, with no evidence of regional or distant spread [7]. Recurrence was defined as a subsequent manifestation of CMM within ten years of initial diagnosis and treatment. In addition, the disease-free survival is a period of time in which no CMM recurrence is seen.

## 3. Methods

### 3.1. Search and Selection

**Figure 1:**
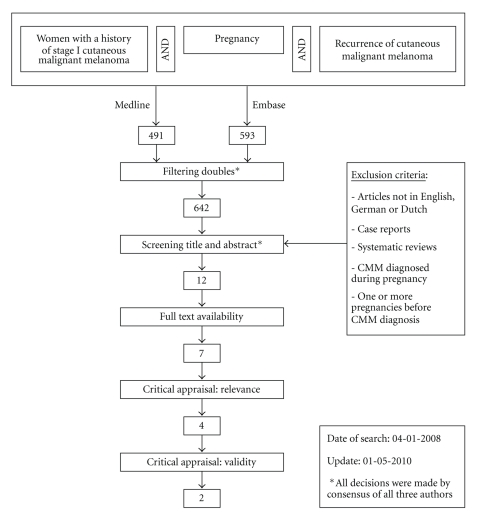
Flow chart.

Synonyms for domain, determinant and outcome were identified using Thesaurus, Embase EMTREE-tool, Medline MesH-terms, and Index [Title/Abstract] and were applied in the electronic databases of Embase and Medline. This full cover search resulted in 491 articles in Medline and 593 articles in Embase (Table 1). The title and abstract of a total of 1084 articles were subsequently screened for their relevance concerning the clinical question, based on the following inclusion criteria: pregnancy within five years of CMM diagnosis in previously nulliparous women, the primary diagnosis being stage I cutaneous malignant melanoma, and CMM recurrence within ten years of the primary diagnosis. The exclusion criteria are shown in the flow chart (Figure 1). All decisions were made through a consensus of all three authors. Upon screening, 12 articles remained for further analysis. A total of seven articles appeared to be full text available. 

### 3.2. Critical Appraisal

The selected articles were screened for relevance of domain, determinant, and outcome. As a result, four articles remained for validity screening. These articles were evaluated on the basis of the components shown in Table 2. Because Shaw [5] and Bork et al. [6] failed to report any effect or precision measures and did not compare patients with controls, these studies were excluded from further assessment. In contrast, Reintgen et al. [9] and MacKie et al. [10] did meet the criteria for a valid study with a level of evidence (Offringa et al. [8]) of 2B and were therefore considered valuable for answering the clinical question.

## 4. Results

Reintgen et al. [9] set up a retrospective case control study, whose objective was to assess whether pregnancy within five years of diagnosis would influence the ten-year disease-free interval in women with stage I cutaneous malignant melanoma. Patients were matched with a control population consisting of female stage I CMM patients between the age of 15 and 44 years, who were not pregnant either at diagnosis or within five-years of diagnosis. The study showed that the ten-yeardisease-free interval of the patient group did not significantly differ from that of the control group (Table 3).

MacKie et al. [10] also established a retrospective case control study, in which the effect of pregnancy on the twenty year-disease-free survival after diagnosis of stage I cutaneous malignant melanoma was investigated. Patients were matched with a control population of women who had completed all pregnancies before a stage I CMM was diagnosed. The study yielded a relative risk of 1.21, which would suggest a slight effect of pregnancy on CMM recurrence. After regression analysis however, this did not prove to be significant (Table 3).

## 5. Conclusion

According to both Reintgen et al. [9] and MacKie et al. [10], pregnancy does not increase the recurrence risk of cutaneous malignant melanoma in women with a history of stage I cutaneous malignant melanoma.

## 6. Discussion

This evidence-based case report suggests that there is no negative influence of pregnancy on the recurrence of cutaneous malignant melanoma in women. There are however a number of drawbacks with respect to the critically appraised studies. Firstly, both Reintgen et al. [9] and MacKie et al. [10] used only a small number of patients compared to the relatively large control groups that were included. Secondly, in the study of MacKie et al. [10] the results of the patient group were not compared with the results of the control group, but both groups were compared with women diagnosed with a CMM in between pregnancies. Reintgen et al. [9] on the other hand used a more realistic control group composed of nulliparous women with a CMM diagnosis. Worth mentioning is the use of a regression analysis in both the study of Reintgen et al. [9] and that of MacKie et al. [10], which is a reliable statistical analysis method to determine the effect of possible confounders.

## 7. Recommendation

The best available evidence does not show any effect of pregnancy on the recurrence risk of cutaneous malignant melanoma in women. Nevertheless, a careful recommendation should be given, with Breslow thickness, Clark level, and skin localization taken into account. Because recurrence, independent of pregnancy, is most likely to develop within two to three years after CMM diagnosis and treatment [11, 12], and since both diagnostic and therapeutical interventions are possibly harmful to the unborn child, appropriate timing is necessary. Women should therefore consider delaying pregnancy for at least two to three years, taking into account the concomitant risk of maternal age related problems such as infertility and fetal developmental abnormalities.

## Figures and Tables

**Table 1:** Search strategy.

Database	Search^a^	Hits
Medline	((Woman OR Women OR Female OR Females OR Patient OR Patients) AND (Skin OR Cutaneous OR Malignant OR Melanoma OR Melanomas)) AND (Pregnancy OR Pregnancies OR Pregnant OR Gravidity OR Gravidities OR Gestation) AND (Recurrent OR Recurrence OR Recurrences OR Recurring OR Recidive OR Recidives OR Recidivism OR Recidivisms OR Recidivating OR Recidivation OR Relapsing OR Relapse OR Relapses)	491

Embase	Idem Medline	593

^**a**^The original search was performed on 04-01-2008 according to the strategy above.

An update performed on 01-05-2010 following the same search strategy yielded no additional relevant articles.

**Table 2:** Critical appraisal.

Study	Study design	Relevance			Blinding			Missing Data		Internal validity					Results	
		Domain	Determinant	Outcome	Determinant	Outcome	Follow-up (months)	Drop-out (%)	Loss-to-follow-up (%)	Selectionbias	Informationbias	External Validity	Standardisation	Level of Evidence [8]	Effect measures	Precision measures
Bork et al. [6]	Retrospective	Women in the childbearing years with stage I malignant melanoma.	Pregnancy after primary therapy.	Recurrence & Disease-free survival.	n.a.	n.a.	n.a.	?	?	n.a.	n.a.	−	−	3	None	None

Reintgen et al. [9]	Retrospective case/control	Women in the childbearing years treated for stage I primary cutaneous melanoma.	Pregnancy within 5 years of diagnosis.	Disease-free interval (10 years).	n.a.	n.a.	n.a.	None	None	n.a.	n.a.	+	±	2B	*χ* ^2^	*P*

Shaw et al. [5]	Retrospective	Women with malignant melanoma.	Pregnancy after diagnosis.	Local recurrence.	n.a.	n.a.	n.a.	?	?	n.a.	n.a.	−	?	3	None	None

MacKie et al. [10]	Retrospective cohort	Women in the reproductive years treated for stage I primary melanoma.	First pregnancy after diagnosis.	Disease-free survival (20 years).	n.a.	n.a.	n.a.	None	None	n.a.	n.a.	+	±	2B	RR	p&CI

+: Good, ±: Moderate, −: Poor, ?: Unknown, n.a.: Not Applicable, RR: Relative risk, CI: Confidence Interval, *P*: *P*-value.

**Table 3:** Influence of pregnancy on recurrence of cutaneous malignant melanoma in women.

Study	Population (*n*)	Effect		Precision	
		*χ* ^2^(*)	RR	*P*-value	95% CI
Reintgen et al. [9]	43 patients	0.04 univariate	—	.80 univariate	—
	585 controls	0.0 multivariate	—	1.00 multivariate	—

MacKie et al. [10]	85 patients	—	1.21 (**)	.66	0.52–2.79
	143 controls	—	0.71 (**)	.54	0.23–2.15

(*) With *χ* ^2^, the chi-square test is pronounced, which was used to test the significance of differences between the patient and the control groups. A concomitant *P*-value >  .05 means that a correlation between pregnancy and CMM recurrence is not likely, and that if there is a correlation at all, this is not very strong, as suggested by the small *χ* ^2^ -values of 0.04 and 0.0 in the univariate and multivariate analyses, respectively.

(**) Compared to women diagnosed with a CMM in between pregnancies.
